# Unhealthy fat distribution as a sex-specific predictor of declining hippocampus insulin sensitivity

**DOI:** 10.1007/s00125-026-06787-2

**Published:** 2026-07-03

**Authors:** Leontine Sandforth, Ralf Veit, Jürgen Machann, Corinna Dannecker, Gregor Miller, Marina Jiménez-Muñoz, Andreas Peter, Reiner Jumpertz von Schwartzenberg, Andreas Fritsche, Martin Heni, Andreas L. Birkenfeld, Hubert Preissl, Stephanie Kullmann

**Affiliations:** 1 Internal Medicine IV, Department of Diabetology, Endocrinology and Nephrology, University Hospital Tübingen, Tübingen, Germany; 2 Institute for Diabetes Research and Metabolic Diseases of the Helmholtz Center Munich at the University of Tübingen, Tübingen, Germany; 3 German Center for Diabetes Research (DZD), Munich-Neuherberg, Germany; 4 Section on Experimental Radiology, Department of Diagnostic and Interventional Radiology, Eberhard Karls University, Tübingen, Germany; 5 Core Facility Statistical Consulting, Helmholtz Center Munich, Munich, Germany; 6 Institute for Clinical Chemistry and Pathobiochemistry, Department for Diagnostic Laboratory Medicine, University Hospital of Tübingen, Tübingen, Germany; 7 M3 Research Center (Malignom, Metabolome, Microbiome), Tübingen, Germany; 8 Cluster of Excellence EXC 2124 ‘Controlling Microbes to Fight Infections’ (CMFI), University of Tübingen, Tübingen, Germany; 9 Division of Endocrinology and Diabetology of Internal Medicine I, Ulm University, Ulm, Germany; 10 Cardiovascular Medicine & Sciences, King’s College London, London, UK; 11 Institute of Pharmaceutical Sciences, Department of Pharmacy and Biochemistry, Interfaculty Centre for Pharmacogenomics and Pharma Research at the Eberhard Karls University Tübingen, Tübingen, Germany

**Keywords:** Functional brain imaging, Hippocampus, Insulin resistance, Menopause, Neurodegeneration

## Abstract

**Aims/hypothesis:**

Impairments in peripheral glucose metabolism and reduced brain insulin sensitivity are linked to an increased risk of both metabolic and neurodegenerative diseases. Brain insulin resistance represents a shared pathological mechanism underlying these disorders. Notably, hippocampal insulin responsiveness declines with age and differs between men and women. This study aimed to identify clinically relevant metabolic predictors of hippocampal insulin sensitivity in the context of age and sex.

**Methods:**

In 260 non-diabetic participants (165 women, mean BMI 29.7 ± 6.2 kg/m^2^, mean age 44.2 ± 16.6 years), functional MRI was performed before and after intranasal insulin administration to assess hippocampal insulin response. Metabolic phenotyping comprised laboratory assessments including oral glucose tolerance tests, whole-body MRI and ^1^H-MRS. In addition, participants were assigned to high- and low-risk prediabetes clusters using the Tübingen risk cluster tool. Prediabetes was defined as impaired fasting glucose and/or impaired glucose tolerance and/or elevated HbA_1_c. We used linear regression models to select the most relevant predictors, including interactions with sex and age.

**Results:**

Fasting plasma glucose levels predicted lower hippocampal insulin response with age independently of sex (estimate 0.533, *p*=0.016). Significant interactions were present between age, sex and body fat distribution (waist-to-hip ratio [WHR]: estimate 0.233, *p*=0.010; visceral adipose tissue [VAT]: estimate 0.007, *p*=0.013; intrahepatic lipid content [IHL]: estimate 0.003, *p*=0.010). In women, higher WHR, VAT and IHL were predictors of lower hippocampal insulin responsiveness with increasing age. These effects remained significant after adjusting for BMI. Postmenopausal women showed lower hippocampal insulin responsiveness with higher WHR and IHL (*p*<0.05), and women in high-risk Tübingen prediabetes clusters also showed lower hippocampal insulin responsiveness than men (sex × cluster type: estimate 0.39, *p*=0.02). The hippocampal insulin response did not correlate with hippocampal volume (*p*>0.05).

**Conclusions/interpretation:**

Unhealthy body fat distribution was a sex-dependent predictor for decreased hippocampal insulin sensitivity with increasing age. Older women with high abdominal fat and/or those assigned to high-risk clusters were most vulnerable to impaired insulin responsiveness in the hippocampus. These findings may contribute to explaining sex differences in the development of type 2 diabetes and neurodegenerative diseases.

**Graphical Abstract:**

**Figure:**
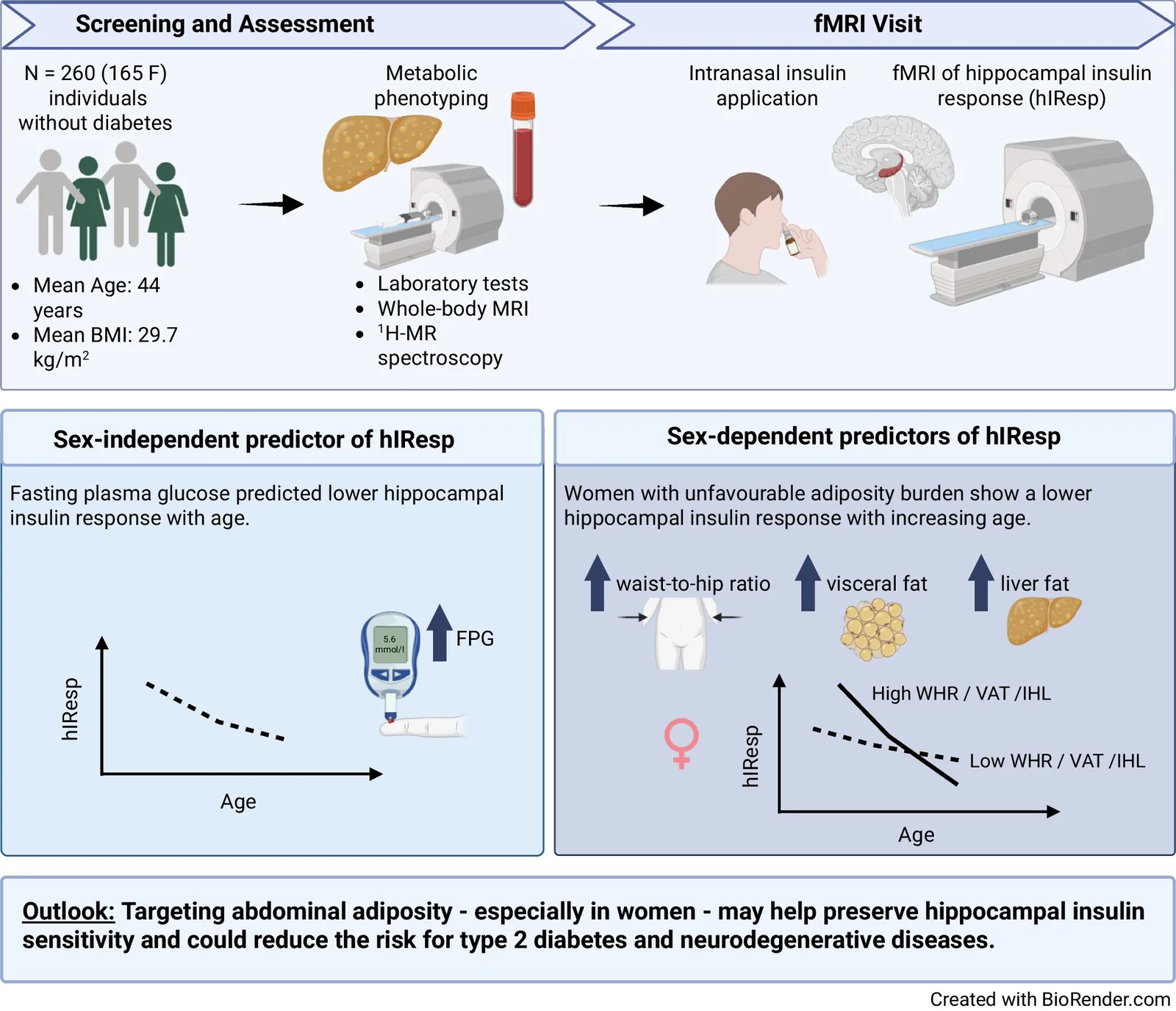

**Supplementary Information:**

The online version contains peer-reviewed but unedited supplementary material available at https://doi.org/10.1007/s00125-026-06787-2.

**Figure:**
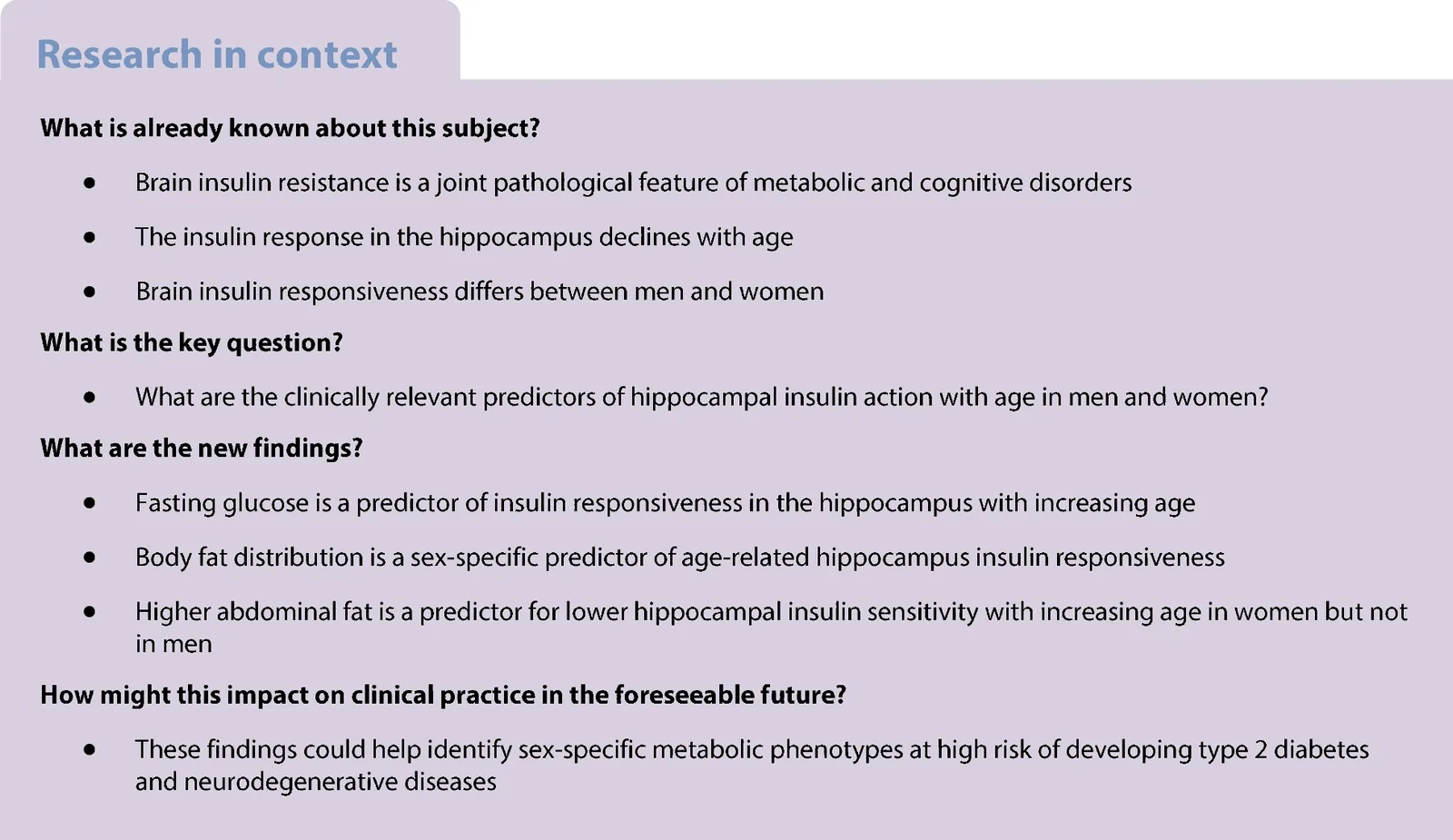

## Introduction

Metabolic disorders such as type 2 diabetes and obesity are amongst the most menacing health threats worldwide, and their prevalence is expected to continue to rise in the years to come [1]. Insulin resistance is a common pathological feature that has traditionally been linked to peripheral organs, such as liver and skeletal muscle. However, the brain plays a central role in energy homeostasis, and has been identified as an insulin-sensitive organ [2]. Brain insulin sensitivity not only influences whole-body glucose metabolism but also body fat distribution [2–4]. In the presence of brain insulin resistance, the reduction of downstream signalling to organs involved in glucose metabolism can potentially influence body fat distribution, leading to ectopic energy storage. Specifically, brain insulin sensitivity was found to predict loss of visceral adipose tissue (VAT), with differences in body fat distribution even 10 years after a lifestyle intervention [4].

Impairments of both peripheral and brain insulin sensitivity may be associated with reduced cognitive functions and with various neurodegenerative diseases [5]. In the healthy state, insulin action in the brain enhances learning and memory, partly through hippocampal function [6]. Preclinical models indicate that hippocampal insulin resistance is a key mediator of cognitive deficits [7].

In addition to its prominent role in memory processes, the hippocampus is sensitive to hormones such as insulin, leptin and ghrelin, thereby mediating and regulating energy homeostasis. The hippocampus has been found to be a critical player in food choice [8–10], with short-term high caloric food intake impairing hippocampal insulin response (hIResp) [6]. Furthermore, steroid hormones may influence hippocampal function, as oestrogen receptors, amongst others, are widely distributed in the brain, with high abundance in the hippocampus. Indeed, central insulin-mediated effects on body weight regulation have been shown to be in part sex-specific, with potentially more pronounced effects on body weight and food intake in healthy men [11]. In young women, central insulin appears to exert more beneficial effects on memory functions [11]. However, the age- and sex-specific differences are not fully understood. The centrally mediated effect of insulin on peripheral insulin sensitivity appears to critically depend on hormonal fluctuations during the menstrual cycle [12], and women tend to show stronger insulin-induced neural food cue reactivity than men [13]. However, with increasing age, women show a stronger decline in brain insulin sensitivity, especially in the hippocampus [14]. Of note, neurodegenerative diseases such as Alzheimer’s disease have been found to be more prevalent in women [15].

In the current study, we investigated metabolic predictors of age-related hIResp in healthy men and women. We used anthropometric and blood-based clinical parameters that characterise metabolic health. In a subset of participants, we additionally investigated MRI-assessed body fat distribution. The risk of developing type 2 diabetes and concomitant diseases may differ markedly between individuals [16]. We therefore assigned a subgroup of participants, who had at least one of the risk factors published by Wagner et al (elevated BMI, family history of type 2 diabetes, prediabetes or history of gestational diabetes) [16], to one of six Tübingen prediabetes clusters (TPCs) [1]. We aimed to identify sex- and age-related associations between hIResp and the TPC.

Brain insulin response was examined by intranasal insulin administration in combination with functional MRI (fMRI) measurements [17]. Administration of insulin intranasally results in rapid transfer across the cribriform plate to the limbic and frontotemporal areas [18]. Human brain-imaging studies have shown that intranasal insulin affects regional blood flow, activity and connectivity in the mesolimbic and cortical regions, including the hippocampus, with subsequent effects on behaviour and peripheral metabolism [2]. Individuals with obesity-associated peripheral insulin resistance, on the other hand, have a disrupted brain response to intranasal insulin, together with a higher preference for palatable foods and altered metabolism [2, 13, 15].

We hypothesised that metabolic predictors related to glucose metabolism, unhealthy body fat distribution and being within a high-risk TPC are linked to a lower hIResp with older age, with stronger age-related effects in women.

## Methods

### Participants

Data acquisition was performed at the University Hospital of Tübingen between 28 January 2013 and 25 October 2022. A dataset of 260 participants without type 2 diabetes was included in the current study. Data were obtained from a database from the Institute for Diabetes Research and Metabolic Diseases (IDM) in Tübingen, Germany, from baseline measurements of several studies under the same conditions to assess brain insulin responsiveness [14]. The source population was of White European ethnicity, from Central Europe (Germany) and across different age groups (18–74 years). Both female and male participants were recruited for the studies; sex was self-reported by the participants. Participants were eligible if they had a BMI of 18–50 kg/m^2^ with normal glucose regulation or prediabetes, and were suitable for MRI measurements. Prediabetes was defined as impaired fasting glucose (5.6–6.9 mmol/l) and/or impaired glucose tolerance (7.8–11.0 mmol/l) and/or elevated HbA_1c_ (39–47 mmol/mol; 5.7–6.4%). Main exclusion criteria included any form of diabetes, centrally acting medication, neurological or psychiatric diseases, and a history of stroke or myocardial infarction. Thus, the sample reflects a relatively healthy cohort with a wide age range. All participants self-identified as White Europeans, which may limit the generalisability of findings on BMI and body fat distribution to other populations [19]. A subset of 206 participants was included for the TPC analysis. The menopausal status of the female participants was determined based on self-report, age greater than 55 years and/or available hormonal measurements (ratio of luteinising hormone to follicle-stimulating hormone significantly below 1 combined with low oestrogen and progesterone levels). Study protocols were approved by the local ethics committees (ethics committees of the Medical Faculty of the Eberhard Karls University and the University Hospital Tübingen), and all experiments were conducted in accordance with the current guidelines. All participants provided written informed consent.

### Experimental design and procedure

All participants underwent a medical examination to rule out psychiatric, neurological or metabolic diseases and to document medication use. After an overnight fast, participants underwent a 75 g oral glucose tolerance test (OGTT) followed by whole-body MRI to assess glucose tolerance and body fat distribution, respectively. The hIResp was quantified in response to intranasal insulin application in combination with fMRI measurements, which were scheduled in the morning after an overnight fast of at least 10 h. After blood sampling, fMRI measurements were recorded under baseline (fMRI1) conditions and 30 min after intranasal insulin spray (fMRI2). Further details have been published previously [14, 20]. Participants received 160 U of human insulin (Actrapid; Novo Nordisk) [17]. The spray was administered over 4 min, with two puffs per nostril every minute. Individuals completed the Food Cravings Questionnaire – Trait (FCQ-T) consisting of 39 items [21] related to eating behaviour on the day of the fMRI measurement. Sum scores were calculated from all items.

### Imaging procedures and processing

#### Brain imaging

Scanning was conducted on 3T whole-body scanners (Magnetom Trio; Siemens Healthcare and Magnetom Prisma, Erlangen, Germany) equipped with 12-channel and 20-channel head coils, respectively. Arterial spin labelling (PASL) images were obtained using a PICORE-Q2TIPS sequence to acquire cerebral blood flow (CBF) before and 30 min after intranasal insulin application. Image preprocessing was performed by using ASLtbx (https://www.nitrc.org/projects/asltbx/) with SPM12 (Wellcome Trust Centre for Neuroimaging, https://www.fil.ion.ucl.ac.uk/spm/docs/installation/) as previously described [14, 22]. CBF values for each measurement were extracted for the bilateral hippocampus and whole grey matter/global volume based on the ‘wfu’ pick atlas (https://www.nitrc.org/projects/wfu_pickatlas). Hippocampal CBF values were normalised by correcting for global CBF differences (CBF_*n*_) to reduce inter-individual and inter-scanner variability. This was done by dividing the CBF values for the hippocampus by individual global CBF values (separately for fMRI1 and fMRI2 measurements). The hIResp was defined as regional CBF change in response to intranasal insulin. For this purpose, normalised CBF maps for each participant were corrected for baseline measurements: ∆CBF_*n*_=CBF_*n*_(fMRI2) – CBF_*n*_(fMRI1).

A high resolution T1-weighted image was used to assess hippocampal and global brain volume. Segmentation of the hippocampus was performed using FreeSurfer image analysis software version 7.4.1 (https://surfer.nmr.mgh.harvard.edu/). Hippocampal volume was normalised by correcting for global brain volume for further analyses.

#### Whole-body MRI for quantification of adipose tissue compartments

Scanning was conducted at a 3T whole-body scanner (Magnetom Vida; Siemens Healthineers, Erlangen, Germany). T1-weighted fast spin-echo images of the whole body with a 1 cm slice thickness and an interslice gap of 1 cm were obtained (as described previously [23]). The VAT and subcutaneous adipose tissue (SAT) in the trunk were segmented applying a fuzzy-clustering algorithm as described previously [23]. To quantify intrahepatic lipid content (IHL), single-voxel MRS was performed in the posterior part of segment 7 of the liver, and used to calculate the ratio of lipid signal and water plus lipid signal (as described previously [24]).

### Analytical procedures for blood samples

Laboratory analyses were performed as previously described [20].

### Clustering

Participants were assigned to TPCs as described by Wagner et al [16]. The clinical variables used for clustering were BMI, hip and waist circumference, fasting glucose and insulin, 2 h glucose and insulin during OGTT, fasting triglycerides and HDL-cholesterol levels. The low-risk clusters 1, 2 and 4 and the high-risk clusters 3, 5 and 6 are grouped here into ‘low-risk clusters’ (*n*=93, 45 women) and ‘high-risk clusters’ (*n*=113, 73 women), respectively. Fifty participants were not assigned to any cluster due to missing data or missing the risk factor information necessary to qualify for this clustering approach. Individuals in the high-risk clusters (clusters 3, 5 and 6) are characterised by a high risk of developing type 2 diabetes, nephropathy, cardiovascular disease and/or have high mortality risk [16]. By contrast, individuals in the low-risk clusters (clusters 1, 2 and 4) have a relatively low risk for type 2 diabetes and cardiometabolic diseases.

### Statistical analyses

Statistical analyses were performed using R for macOS with R studio (version 4.2.2; Cran R Project, https://cran.r-project.org/bin/macosx/; Posit, https://posit.co/download/rstudio-desktop). The baseline characteristics of female and male participants were compared using the Mann–Whitney *U* test for continuous variables and the χ^2^ test for categorical variables (Table 1).

**Table 1:** Metabolic and behavioural characteristics of study participants

	Female (*n*=165)	Male (*n*=95)	*p* value
Age (years)			
Mean ± SD	43.0 ± 16.8	45.4 ± 16.1	0.172
Median [Q1–Q3]	44.0 [26.0–59.0]	46.0 [30.0–60.5]	
BMI (kg/m^2^)			
Mean ± SD	29.7 ± 6.9	29.8 ± 4.8	0.592
Median [Q1–Q3]	29.8 [23.6–33.1]	29.3 [25.9–32.4]	
WHR			
Mean ± SD	0.84 ± 0.07	0.96 ± 0.06	<0.001
Median [Q1–Q3]	0.84 [0.78–0.89]	0.96 [0.91–0.99]	
TPC category			
High-risk cluster (3, 5, 6)	73 (44.2)	40 (42.1)	<0.001
Low-risk cluster (1, 2, 4)	45 (27.3)	48 (50.5)	
No cluster assigned	47 (28.5)	7 (7.4)	
Glycaemic category			
IFG and/or IGT	45 (27.3)	40 (42.1)	0.017
Normal glucose tolerance	120 (72.7)	54 (56.8)	
No category assigned	0	1 (0.1)	
FPG (mmol/l)			
Mean ± SD	5.1 ± 0.5	5.3 ± 0.6	0.004
Median [Q1–Q3]	5.0 [4.8–5.4]	5.2 [4.9–5.6]	
Fasting plasma insulin (pmol/l)			
Mean ± SD	82.5 ± 48.5	83.8 ± 58.7	0.574
Median [Q1–Q3]	70.0 [51.0–100.0]	68.5 [49.0–106.0]	
Fasting C-peptide glucose ratio			
Mean ± SD	5.7 ± 2.2	5.7 ± 2.3	0.896
Median [Q1–Q3]	5.4 [4.0–7.0]	5.3 [4.0–6.9]	
2 h plasma glucose (mmol/l)			
Mean ± SD	6.2 ± 1.5	6.4 ± 1.8	0.578
Median [Q1–Q3]	5.9 [5.1–7.2]	6.0 [5.0–7.7]	
2 h plasma insulin (pmol/l)			
Mean ± SD	460.0 ± 360.0	500.0 ± 560.0	0.221
Median [Q1–Q3]	340.0 [240.0–580.0]	300.0 [150.0–620.0]	
HbA_1c_ (mmol/mol)			0.248
Mean ± SD	36.2 ± 3.7	36.9 ± 4.3	
Median [Q1–Q3]	35.5 [33.3–38.8]	36.6 [33.3–39.9]	
HbA_1c_ (%)			
Mean ± SD	5.5 ± 0.3	5.5 ± 0.4	0.248
Median [Q1–Q3]	5.4 [5.2–5.7]	5.5 [5.2–5.8]	
Triglycerides (mmol/l)			
Mean ± SD	1.2 ± 0.7	1.5 ± 1.2	0.198
Median [Q1–Q3]	1.1 [0.8–1.5]	1.2 [0.8–1.8]	
HDL-cholesterol (mmol/l)			
Mean ± SD	1.5 ± 0.4	1.3 ± 0.4	<0.001
Median [Q1–Q3]	1.5 [1.2–1.8]	1.2 [1.0–1.5]	
VAT volume (l), *n*=180			
Mean ± SD	3.5 ± 1.8	5.7 ± 2.8	<0.001
Median [Q1–Q3]	3.4 [2.2–4.6]	5.6 [3.4–7.6]	
IHL (%), *n*=177			
Mean ± SD	5.6 ± 5.5	8.2 ± 7.8	0.030
Median [Q1–Q3]	3.5 [1.9–7.4]	5.6 [2.3–11.3]	
SAT volume (l), *n*=180			
Mean ± SD	18.2 ± 6.1	13.0 ± 3.8	<0.001
Median [Q1–Q3]	18.1 [13.8–21.1]	12.8 [10.7–14.9]	
Menopausal status			
Postmenopausal	68 (41.2)	0	
Premenopausal	97 (58.8)	0	
Not applicable	0	95 (100)	
FCQ-T score			
Mean ± SD	95.4 ± 29.2	83.9 ± 25.4	0.004
Median [Q1–Q3]	97.5 [73.0–116.0]	81.0 [64.0–99.0]	

Values for categorical variables are *n* (%)

*p* values are the result of testing differences between the two sex groups using either the Mann–Whitney *U* test for continuous, non-normally distributed variables, a simple unpaired *t* test for continuous normally distributed variables, or the χ^2^ test for categorical variables

IFG, impaired fasting glucose; IGT, impaired glucose tolerance

To identify the most relevant metabolic predictors among anthropometric and blood-based clinical parameters, we first performed stepwise linear regression to model hIResp using the Bayesian information criterion [25]. This included sex, age and a third metabolic component (including BMI, waist-to-hip ratio [WHR], fasting plasma glucose [FPG], 2 h glucose, the Matsuda insulin sensitivity index, HbA_1c_, fasting triglyceride level and the C-peptide/glucose ratio). Collinearity was assessed using the generalised variance inflation factor (GVIF) (electronic supplementary material [ESM] Table 1). Second, we used separate two-sided linear regression models to examine the influence of different body fat compartments on hIResp, including interactions with sex and age (separate three-way interactions for each body fat compartment). These models included SAT volume, VAT volume and IHL. Third, we used two-sided linear regression to examine the relationship between hIResp and low- and high-risk TPCs. Fourth, we analysed correlations of global brain volume and normalised hippocampal volume with age, metabolic predictors and hIResp separately in men and women. Correlations were reported using Pearson’s correlation coefficient. Finally, we examined the influence of sex and age on food craving assessed using the FCQ-T in a linear regression model.

To visualise interactions between continuous variables, plots were generated using equally sized groups based on sex-specific quartiles, as grouping was required to display interactions involving three continuous variables. Simple slope analyses within these equally sized groups were then performed to further characterise significant interactions identified in the selected models using the ‘emtrends’ function from the ‘emmeans’ package in R Studio. Simple slope estimates are reported with 95% CIs to evaluate significance. A two-sided *p* value of <0.05 was considered statistically significant for all analyses. Bonferroni correction was applied as appropriate according to the number of variables or tests performed; no additional correction for multiple testing was applied to the stepwise regression models, as selection of variables was based on the Bayesian information criterion. Residual normality was assessed using Q–Q plots.

## Results

Datasets from 165 women and 95 men (mean BMI 29.7 ± 6.2 kg/m^2^, range 18.2–49.5 kg/m^2^; mean age 44.2 ± 16.6 years, range 18–74 years) were included in the current study. Metabolic and behavioural characteristics are shown in Table 1.

### FPG and WHR predict the hIResp

First, we examined metabolic predictors related to glucose metabolism and body fat distribution in relation to the hIResp using stepwise linear regression (Table 2 and ESM Table 2). These included sex, age, BMI, WHR, FPG, 2 h glucose, Matsuda insulin sensitivity index, HbA_1c_, fasting triglyceride level and the C-peptide/glucose ratio.

**Table 2:** Stepwise linear regression model to identify the most relevant predictor of hippocampal insulin responsiveness

Variable	Estimate	Standard error	*t* value	*p* value
Intercept	−4.668	1.716	−2.720	0.007
Sex	7.875	3.679	2.140	0.033
Age	0.117	0.039	3.024	0.003
WHR	2.878	1.932	1.490	0.138
FPG	0.533	0.219	2.429	0.016
Sex × age	−0.206	0.083	−2.477	0.014
Sex × WHR	−9.000	4.015	−2.241	0.026
Age × WHR	−0.080	0.045	−1.784	0.076
Age × FPG	−0.012	0.004	−2.596	0.010
Sex × age × WHR	0.233	0.089	2.614	0.010

Coefficients of selected model using stepwise linear regression for hIResp, using the Bayesian information criterion to select the most relevant predictors of metabolic health among anthropometric and blood-based clinical parameters (*n*=258, one missing value for FPG and one missing value for WHR). Effect size-adjusted *R*=8 × 10^−2^

**Fig. 1:**
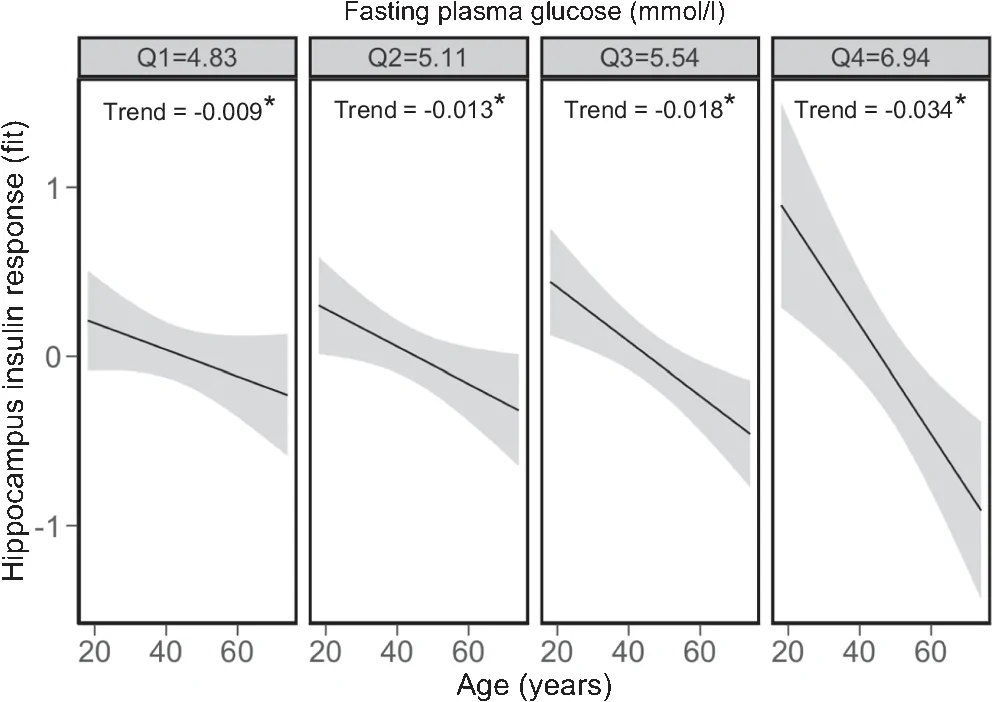
Age-dependent hIResp with increasing FPG in female and male participants. The plots show the effects of a two-way interaction of FPG × age on the hippocampal response to insulin using a two-sided stepwise linear regression model (*n*=258, one missing value for FPG, one missing value for WHR). Participants with higher FPG show lower insulin responsiveness in the hippocampus with increasing age. The FPG levels were divided into four equally sized groups based on quartiles to display the model results. Shaded areas around the lines indicate 95% CI. Asterisks indicate a significant simple slope, defined as a 95% CI that did not include 0

**Fig. 2:**
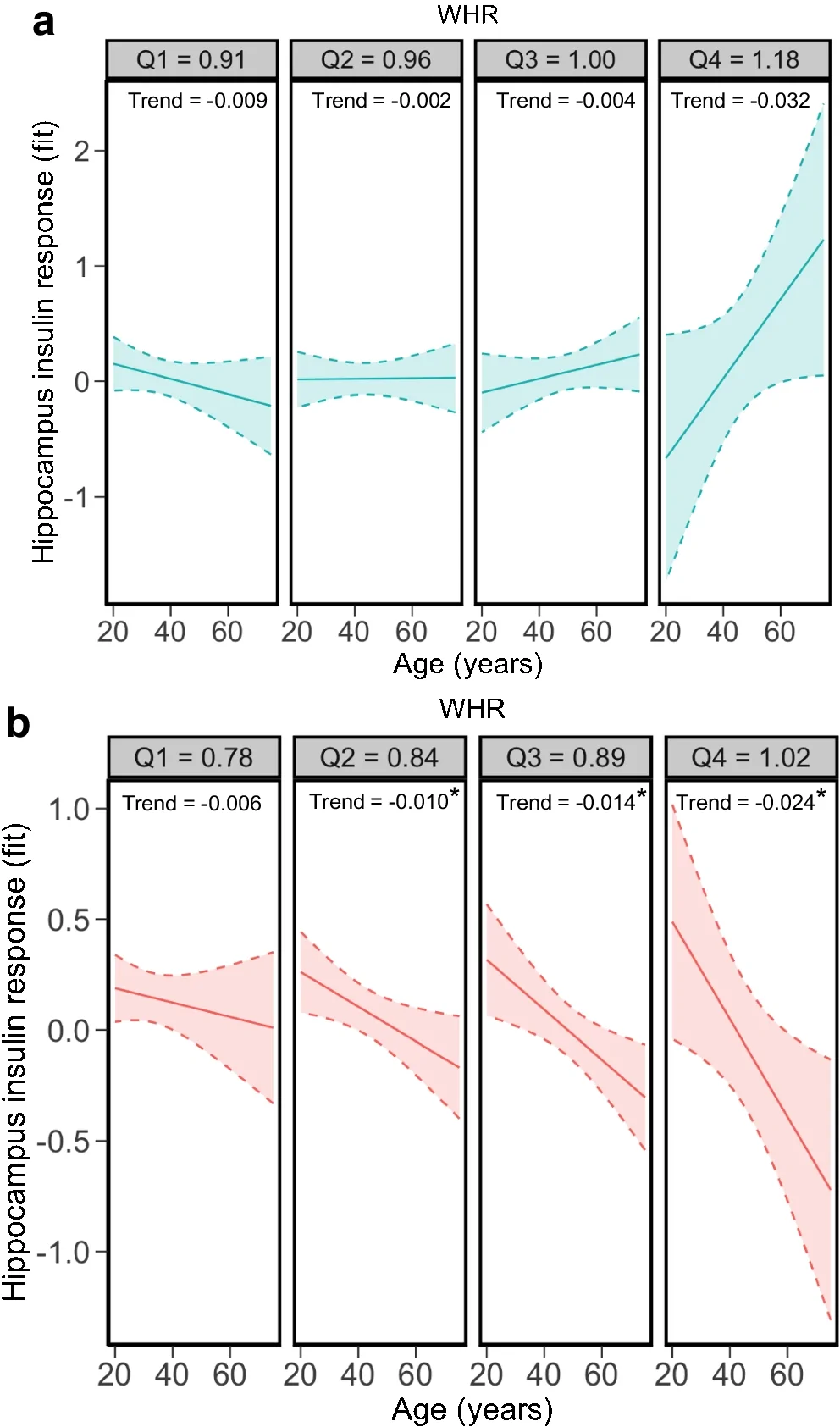
Age-dependent hIResp with increasing WHR in (**a**) male participants and (**b**) female participants. The plots show the effects of the three-way interaction of WHR × age × sex on the hippocampal response to insulin using a two-sided stepwise linear regression model (*n*=258, one missing value for FPG, one missing value for WHR). Female participants with higher WHR show a lower insulin responsiveness in the hippocampus with increasing age. WHR was divided into four equally sized groups based on quartiles to display the model results. Shaded areas around the lines indicate 95% CI. Asterisks indicate a significant simple slope, defined as a 95% CI that did not include 0

Sex, age and FPG were significant predictors of hIResp. Significant two-way interactions were found between sex and age, sex and WHR, and age and FPG (*p*<0.05, Table 2; results adjusted for BMI are shown in ESM Table 3). Simple slope analysis showed a lower age-dependent hIResp in individuals with higher FPG, independent of sex (Fig. 1 and ESM Table 4; results adjusted for BMI are shown in ESM Table 5). Moreover, a significant three-way interaction was observed between sex, age and WHR (*p*=0.01; Table 2 and Fig. 2). Slope analysis showed that older women with a higher WHR had the lowest hIResp (Fig. 2 and ESM Table 6; results adjusted for BMI are shown in ESM Table 7).

### Higher VAT and IHL predict the hIResp in a sex-dependent manner

**Fig. 3:**
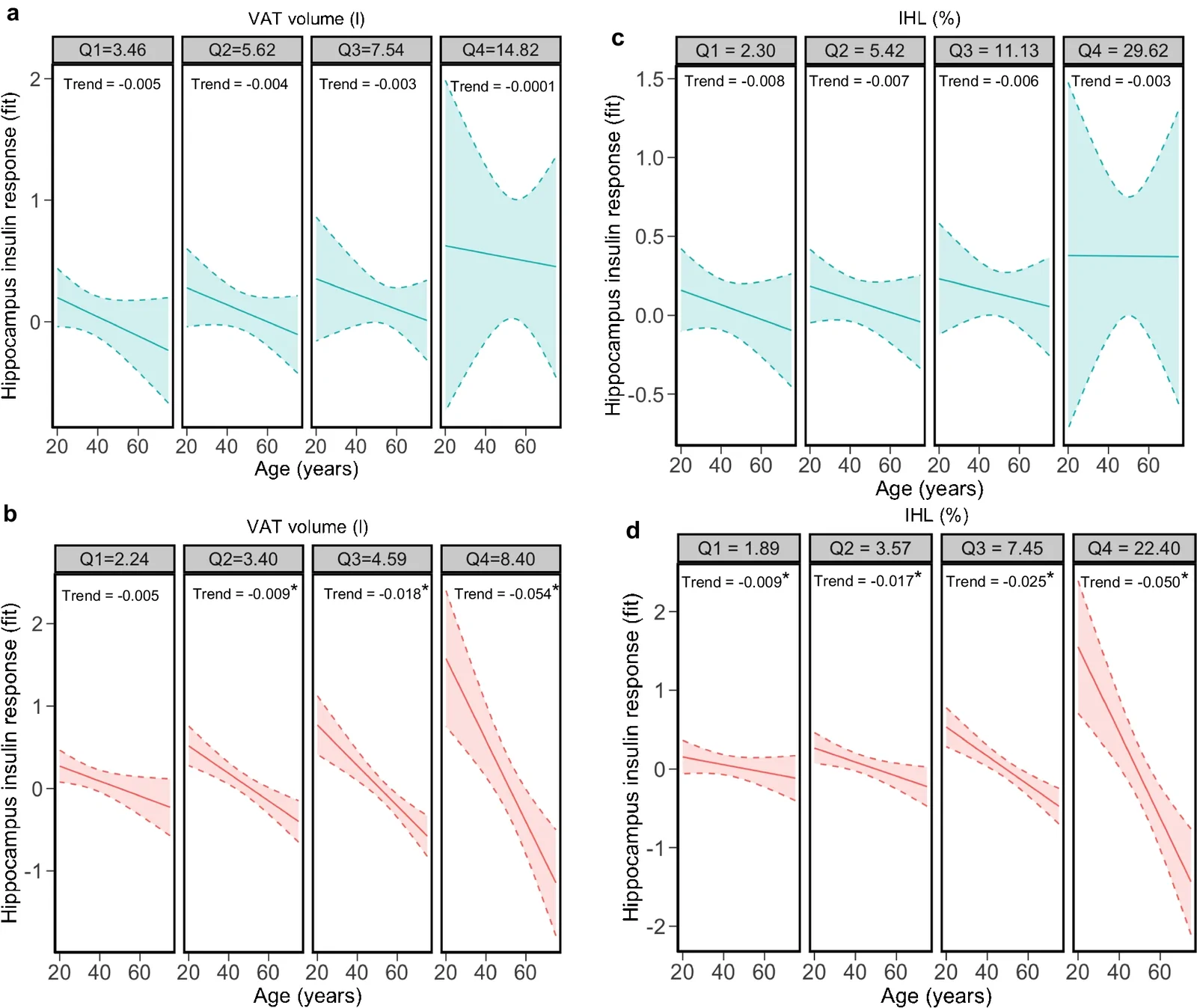
Age-dependent hIResp with increasing VAT in (**a**) male participants and (**b**) female participants; and with increasing IHL in (**c**) male participants and (**d**) female participants. The plots show the effects of the three-way interaction of VAT × age × sex and IHL × age × sex, respectively, in relation to the hippocampal response to insulin using a two-sided linear regression model (*n*=180, 80 missing values for VAT; *n*=177, 83 missing values for IHL). Female participants with higher VAT or IHL show a lower insulin response in the hippocampus with increasing age. VAT and IHL were divided into four equally sized groups based on quartiles to display the model results. Shaded areas around the lines indicate 95% CI. Asterisks indicate a significant simple slope, defined as a 95% CI that did not include 0

Next, we examined the associations of whole-body MRI-derived fat compartments (SAT, VAT and IHL) with hIResp, including sex and age as interaction terms in all models. To account for multiple testing across the three fat compartments, statistical significance was set at *p*<0.016 (Bonferroni correction: 0.05/3). Significant three-way interactions were observed between VAT, sex and age (*p*=0.013; Table 3, Fig. 3a, b, ESM Table 8) and between IHL, sex and age (*p*=0.010; Table 4, Fig. 3c, d, ESM Table 9) in relation to hIResp. These effects remained significant after adjusting for BMI (ESM Tables 10 and 11). Slope analysis showed a negative association of age-dependent hIResp with VAT and IHL in women but not in men (ESM Tables 12–15). SAT was not a significant predictor of hIResp (*p*>0.05, ESM Tables 16 and 17).

**Table 3:** Linear regression model to examine the influence of VAT on the hIResp

Variable	Estimate	Standard error	*t* value	*p* value
Intercept	−0.314	0.314	−1.000	0.319
Sex	0.571	0.532	1.074	0.285
Age	0.006	0.008	0.740	0.460
VAT	0.343	0.105	3.254	0.001
Sex × age	−0.015	0.012	−1.225	0.222
Sex × VAT	−0.314	0.142	−2.213	0.028
Age × VAT	−0.007	0.002	−3.096	0.002
Sex × age × VAT	0.007	0.003	2.522	0.013

Coefficients of the two-sided linear regression model for hIResp, and the interactions between sex, age and VAT (*n*=180, 80 missing values for VAT). Effect size-adjusted *R*^2^=1.2 × 10^−1^

**Table 4:** Linear regression model to examine the influence of IHL on the hIResp

Variable	Estimate	Standard error	*t* value	*p* value
Intercept	0.031	0.216	0.142	0.887
Sex	0.211	0.344	0.613	0.541
Age	0.000	0.005	−0.069	0.945
IHL	0.116	0.035	3.291	0.001
Sex × age	−0.005	0.008	−0.619	0.537
Sex × IHL	−0.112	0.050	−2.222	0.028
Age × IHL	−0.002	0.001	−3.524	0.001
Sex × age × IHL	0.003	0.001	2.606	0.010

Coefficients of the two-sided linear regression model for hIResp, and the interactions between sex, age and IHL (*n*=177, 83 missing values for IHL). Effect size-adjusted *R*^2^=1.2 × 10^−1^

In an exploratory analysis, we evaluated whether the relationship between body fat distribution and hIResp depends on menopausal status in women. We observed significant interactions of menopausal status with WHR, VAT and IHL (WHR × menopausal status: *p*=0.003, ESM Fig. 1a; VAT × menopausal status: *p*=0.007, ESM Fig. 1b; IHL × menopausal status: *p*<0.001, ESM Fig. 1c). Based on the slope analysis, postmenopausal women with higher WHR and IHL showed the lowest hIResp; this relationship was not observed for VAT in postmenopausal women (ESM Table 18). However, in premenopausal women, higher VAT and IHL were associated with a higher hIResp (ESM Fig. 1B, C and ESM Table 18). When this was adjusted for BMI, a positive association remained significant only for IHL and menopausal status (ESM Table 19).

### TPCs interact with the hIResp in a sex-dependent manner

**Fig. 4:**
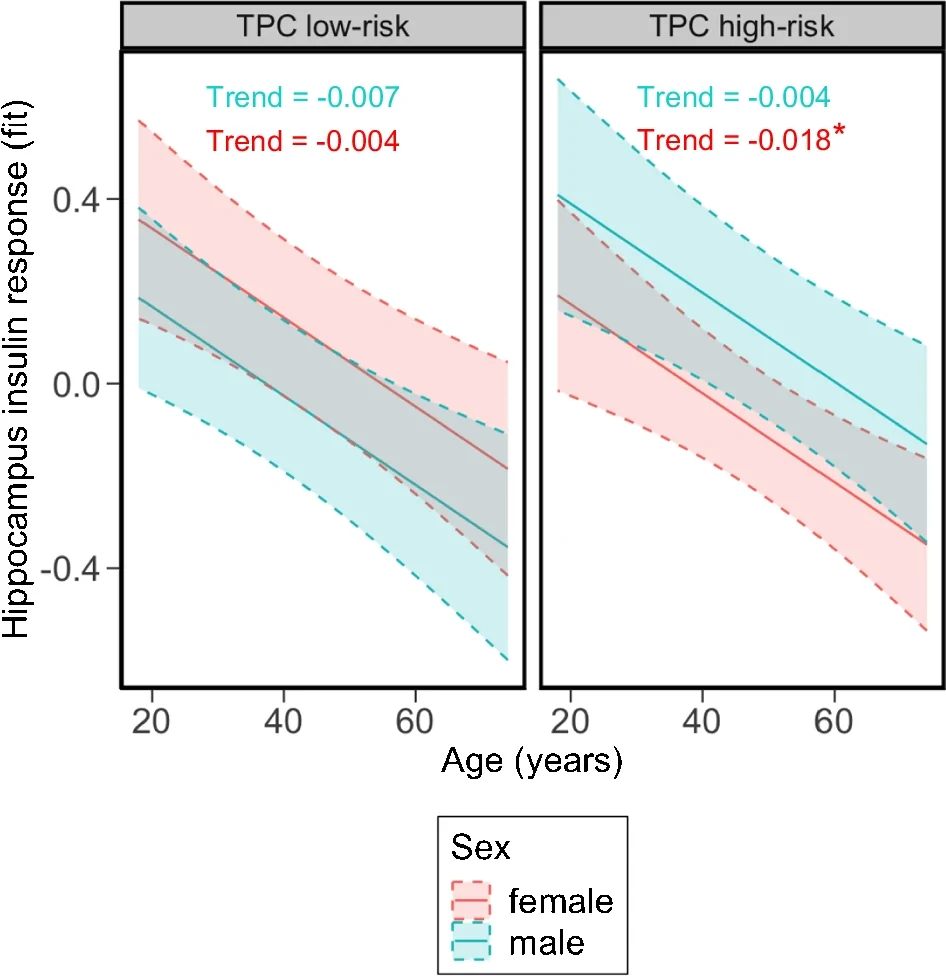
Age-dependent hIResp in low and high-risk TPCs in male participants (green) and female participants (red). Low risk comprises clusters 1, 2 and 4; high risk comprises clusters 3, 5 and 6. The plots show the effects of an exploratory two-way interaction of cluster type × sex in relation to the hippocampal response to insulin using a two-sided linear regression model (*n*=206). Shaded areas around the lines indicate 95% CI. Asterisks indicate a significant simple slope, defined as a 95% CI that did not include 0

In an exploratory analysis, we evaluated hIResp according to the TPC allocation. Recent findings point to the existence of specific risk subgroups amongst individuals who are at risk for type 2 diabetes [16]. The interaction between cluster type and sex was a significant predictor for hIResp (sex × cluster type interaction: estimate 0.39, standard error 0.166, *t* value 2.339, *p*=0.02; Fig. 4 and ESM Table 20). A further exploratory subgroup analysis of the TPCs indicated that this effect may be driven by women in high-risk cluster 6 (ESM Fig. 2 and ESM Table 21). No correction for multiple testing was applied.

### Normalised hippocampal volume does not correlate with hIResp

Brain atrophy is a characteristic feature of Alzheimer’s disease. Moreover, lower brain and hippocampal volumes have been reported in individuals with obesity with increasing age, and structural brain differences between men and women have been reported throughout the lifespan [26]. To account for multiple testing, statistical significance was set at *p*<0.0083 (Bonferroni correction for number of correlations performed: 0.05/6). In this study, global brain volume was not associated with age, although a trend was observed in men (men: *R*=−0.33, *p*=0.02; women: *R*=−0.14, *p*=0.16; ESM Fig. 3a). Normalised hippocampal volume was not associated with age in men (*R*=−0.26, *p*=0.07) or in women (*R*=−0.16, *p*=0.12) (ESM Fig. 3b). Apart from age, the metabolic predictors identified as associated with hIResp did not correlate with hippocampal volume (*p*>0.05). Furthermore, hIResp did not correlate with hippocampal volume (*p*>0.05).

### Women and men differ in food craving according to age

Previous studies reported higher food craving in individuals with insulin resistance, and the hIResp has been shown to predict desire to eat [27]. Age significantly predicted the reported food craving in a sex-dependent manner (interaction sex × age: estimate −0.48, standard error 0.235, *t* value −2.059, *p*=0.04; ESM Table 22), such that women and men differed in food craving with age (ESM Fig. 4). Simple slope analysis did not confirm a significant relationship between food craving and age (ESM Table 23), and none of the metabolic predictors including body fat distribution or hIResp predicted food craving (*p*>0.05).

## Discussion

In the current study, we evaluated clinically relevant metabolic parameters of the hIResp. Independently of sex, fasting glucose levels predicted lower hIResp with increasing age in individuals without type 2 diabetes. Unhealthy body fat distribution was a sex-dependent predictor of hIResp. Older women with high WHR, VAT and/or IHL showed the lowest hIResp, and women had higher food craving with age than men. Furthermore, women assigned to the high-risk TPCs showed lower hIResp with age, a relationship that was not significant in men.

The hippocampus is involved in memory processes, imagination and scene recognition, but also has a pronounced role in physiological regulation, including food intake. In rodent models, distinct hippocampal neural populations control hedonic and macronutrient-specific food intake [8], and central insulin promotes cell survival and synaptic plasticity in the hippocampus as well as hippocampus-dependent memory [28]. Recent imaging studies in humans show that insulin action in the hippocampus is reflected by increased regional blood flow and functional connectivity, which predict a subsequent decrease in the desire to eat [13, 27]. Moreover, a high caloric diet induced lower hIResp in healthy young men prior to changes in glucose metabolism [6]. In the current study, FPG was a sex-independent predictor of age-related hIResp. Previous studies have shown that higher FPG is associated with structural alterations in the hippocampus as well as lower memory function [29, 30]. We show here that regional hippocampal blood flow did not increase in response to insulin in older women with higher VAT and IHL; these women also report more food cravings. With increasing age, hIResp has been shown to decrease, particularly in women [14]. Moreover, a higher WHR in midlife is associated with lower hippocampal functional connectivity later in life [31]. In the current study, we were able to specify metabolic predictors in older women at increased risk for lower hIResp. In line with this, post-mortem brain tissue of individuals with Alzheimer’s disease has been found to show impaired hippocampal insulin signalling, partly independent of peripheral insulin resistance [32]. Importantly, our results were not driven by brain or hippocampal atrophy, which are typical markers of Alzheimer’s disease. This aligns with recent findings suggesting that age-related brain volume loss does not explain the greater prevalence of Alzheimer’s disease in women [33]. Hence, hippocampal insulin resistance could be a potential link and an early marker for women at high risk of developing metabolic and cognitive diseases [26] that may emerge prior to alterations in glucose metabolism and cognitive decline.

Due to the differential exposure to oestrogen and testosterone in men and women, brain ageing may not affect men and women equally. Furthermore, studies in premenopausal women have described the impact of sex hormones on brain insulin responsiveness. One recent study demonstrated a stronger brain insulin effect in the follicular phase of the menstrual cycle [12], which may reflect the role of oestradiol in brain insulin sensitivity, as previously shown in lean but not obese rats [34]. However, behavioural studies in humans did not observe effects of oestradiol on memory after intranasal insulin administration [35]. Recent data on the influence of the menstrual cycle on hippocampal functional connectivity may point to the impact of progesterone [36]. However, the mechanisms by which sex hormonal fluctuations over the lifespan affect brain insulin action require further investigation.

Our data show that body fat distribution appears to be differentially associated with hIResp and ageing in women and men. In women, higher WHR, VAT and IHL were associated with a lower hIResp at higher age, whereas SAT did not predict hIResp. Previously, body fat distribution and the associated metabolic risk have been shown to differ markedly between women and men. Women are often diagnosed with type 2 diabetes at a higher BMI than men are [37]. One explanation could be that, especially prior to menopause, women tend to store more fat as SAT, particularly in the lower extremities. Men are prone to have relatively more VAT, which is generally associated with an elevated metabolic risk. During and after menopause, the primary source of oestrogen in women shifts from the ovaries to peripheral tissues, especially adipose tissue, where androgens are converted to oestrogen. After the menopause, women undergo a marked shift in body fat distribution linked to hormonal changes [38], characterised by increased VAT and liver fat [39], which is probably related to oestrogen deficiency, VAT redistribution, and more severe insulin resistance [39]. High abdominal fat may therefore reflect advanced metabolic deterioration, and both result from and contribute to an unfavourable hormonal milieu. In the current study, postmenopausal women showed a significantly lower hIResp with higher WHR and liver fat, whereas premenopausal women showed higher hIResp with higher VAT and liver fat. Further studies are needed to clarify how sex hormones and ageing shape the relationship between body fat distribution and hIResp in women. In the current study, we found that women assigned to the high-risk TPCs, particularly cluster 6, showed decreased hIResp. High-risk TPCs are characterised, among other features, by high insulin resistance, unfavourable body fat distribution and a high risk of developing type 2 diabetes and related comorbidities, as well as higher mortality risk. Hence, use of TPCs may help identify women at specific risk of age-related hippocampal insulin resistance.

In the future, sex-specific interventions could be an essential tool to overcome brain insulin resistance in the vicious cycle between central and peripheral insulin resistance. Lifestyle and pharmacological interventions have already been shown to normalise brain insulin responsiveness [20, 40]. In particular, aerobic exercise reduced VAT and improved hIResp in middle-aged men and women with obesity [40]. Thus, sex-specific and individually tailored interventions could be beneficial to treat brain insulin resistance. Based on our findings, interventions that specifically target abdominal fat, as in weight loss-induced prediabetes remission [1, 41], should be evaluated for their impact on neurocognitive health. We hypothesise that maintaining hIResp could reduce the risk of development of type 2 diabetes and neurodegenerative diseases in women.

Due to the observed relationship between metabolic and neurocognitive health, diabetes-related drugs could be repurposed for treatment of neurodegenerative diseases. For example, the glucagon-like peptide 1 receptor agonist (GLP1RA) lixisenatide has been shown to decelerate the progression of motor disability in early Parkinson’s disease [42]. In mice, GLP1RAs may reduce neuroinflammation, specifically in the hippocampus, where they may help reduce the risk for neurodegenerative disease [43]. Furthermore, the sodium–glucose transporter 2 inhibitor empagliflozin has been shown to improve brain insulin sensitivity in individuals with prediabetes [20]. These studies add to the evidence demonstrating the close interplay between peripheral metabolism and the brain. Whether targeting brain insulin resistance can improve cognitive health needs to be investigated in future studies.

This study has some limitations. First, data were collected from White Europeans living in the university city of Tübingen (Germany) and the surrounding area, so generalisability to other populations may be limited. Second, the specific impact of sex hormones on hIResp cannot be determined as sex hormone levels were not assessed on the day of fMRI. Third, the explanatory power of the initial models was limited, as indicated by the low adjusted *R*^2^ values. The stepwise regression approach may be sensitive to sample-specific variation, may overestimate the importance of retained predictors, and does not fully account for model uncertainty. Therefore, the identified predictors should be interpreted cautiously and ideally confirmed in independent datasets. Finally, this is a retrospective cross-sectional analysis and, as such, cannot address temporal relationships; prospective, longitudinal studies examining the influence of sex and sex hormones on hIResp are warranted.

In the current study, we show that an unfavourable body fat distribution, previously demonstrated as an indicator for cardiovascular and type 2 diabetes risk, is a substantial predictor for impaired hIResp, particularly in older women. These findings could help identify individuals at risk for neurodegenerative and metabolic diseases, and further elucidate sex differences in central and peripheral metabolism and complications of metabolic diseases. Specifically, women with higher abdominal fat may benefit most from early lifestyle or pharmacological interventions.

## Supplementary Information

Below is the link to the electronic supplementary material.

Supplementary file1 (PDF 1030 KB)

## Data Availability

Data are under the supervision of the Data Access Steering Committee of the Institute for Diabetes Research and Metabolic Diseases; Tübingen, Germany. Data can be made available upon reasonable request in a pseudonymised form. Requests should include a brief research proposal, intended use of the data and research plan. Access is subject to applicable data protection regulations and institutional data-sharing agreements. No custom code was used in this study.

## Abbreviations

CBF: Cerebral blood flow
FCQ-T: Food Cravings Questionnaire – Trait
fMRI: Functional MRI
FPG: Fasting plasma glucose
GLP1RA: Glucagon-like peptide 1 receptor agonist
hIResp: Hippocampal insulin response
IHL: Intrahepatic lipid content
OGTT: Oral glucose tolerance test
SAT: Subcutaneous adipose tissue
TPCs: Tübingen prediabetes clusters
VAT: Visceral adipose tissue
WHR: Waist-to-hip ratio

## Authors’ relationships and activities

RJvS and LS serve as clinical investigators for studies funded by Eli Lilly and Boehringer Ingelheim, and all compensations go to the University Hospital Tübingen. LS receives honoraria from Sciarc GmbH; all compensations go to the University Hospital Tübingen. ALB declines all personal compensation from pharma or device companies; all compensations go to the University Hospital Tübingen. ALB received unrestricted research support from AstraZeneca and Daiichi Sankyo. He has served as principal investigator for studies funded by Novo Nordisk and Boehringer Ingelheim. MH reports lecture fees from Chiesi/Amryt, AstraZeneca, Boehringer Ingelheim, Lilly, Novartis, Novo Nordisk and Sanofi. SK reports lecture fees from Eli Lilly and Novo Nordisk. The remaining authors declare that there are no relationships or activities that might bias, or be perceived to bias, their work.
