# Supplementary material for: Unhealthy fat distribution as a sex-specific predictor of declining hippocampus insulin sensitivity
Source: Diabetologia. 2026 Jul 3;69(10):2957–69. doi: 10.1007/s00125-026-06787-2 (PMC13541856; doi:10.1007/s00125-026-06787-2)
Supplement: Supplementary file 1 [file 125_2026_6787_MOESM1_ESM.pdf]

	GVIF	*df*	GVIF^(1/(2 · *df*)	Interacts With	Other Predictors
**Sex**	132.404	7	1.418	Age, WHR	FPG
**Age**	1.000	9	1.000	Sex, WHR, FPG	0.033
**WHR**	132.404	7	1.418	Sex, Age	FPG
**FPG**	366.912	3	2.676	Age	Sex, WHR

ESM Table 1 Collinearity analysis using the Generalised variance inflation factor (GVIF) for the model. GVIF^(1/(2 · *df*) GVIFs adjusted for degrees of freedom.

	Estimate	Standard error	*t* value	*p* value
**(Intercept)**	-4.668	1.716	-2.720	0.007
**Sex**	7.875	3.679	2.140	0.033
**Age**	0.117	0.039	3.024	0.003
**WHR**	2.878	1.932	1.490	0.138
**FPG**	0.533	0.219	2.429	0.016
**Sex X age**	-0.206	0.083	-2.477	0.014
**Sex X WHR**	-9.000	4.015	-2.241	0.026
**Age X WHR**	-0.080	0.045	-1.784	0.076
**Age X FPG**	-0.012	0.004	-2.596	0.010
**Sex X age X WHR**	0.233	0.089	2.614	0.010

ESM Table 2 Stepwise linear regression model to find the most relevant predictor for hippocampal insulin response (hIResp). Coefficients of selected model using stepwise linear regression hIResp, using the Bayesian Information Criterion to select the most relevant predictors of metabolic health using anthropometric and blood-based clinical parameters (n=258, 1 missing value for FPG and 1 missing value for WHR). Effect size adjusted $R^2 = 8 \cdot 10^{-2}$.

	Estimate	Standard error	*t* value	*p* value
**(Intercept)**	-3.840	1.754	-2.189	0.030
**Sex**	7.264	3.669	1.980	0.049
**Age**	0.100	0.040	2.522	0.012
**WHR**	1.949	1.975	0.987	0.325
**FPG**	0.446	0.222	2.007	0.046
**BMI**	0.013	0.006	2.013	0.045
**Sex X age**	-0.188	0.083	-2.262	0.025
**Sex X WHR**	-8.252	4.008	-2.059	0.041
**Age X WHR**	-0.068	0.045	-1.504	0.134
**Age X FPG**	-0.010	0.004	-2.266	0.024
**Sex X age X WHR**	0.214	0.089	2.393	0.017

ESM Table 3 Coefficients of the stepwise linear regression model for hIResp, using the Bayesian Information Criterion to select the most relevant predictors of metabolic health using anthropometric and blood-based clinical parameters, adjusting for BMI (n=258).

FPG	trend	Standard error	*df*	Lower CL	Upper CL
4.830	-0.009	0.004	248	-0.018	-0.001
5.110	-0.013	0.004	248	-0.021	-0.005
5.540	-0.018	0.005	248	-0.026	-0.009
6.940	-0.034	0.009	248	-0.052	-0.016

ESM Table 4 Simple slope analysis for the results from the stepwise regression model for hIResp with the two-way interaction between FPG and age.

FPG	trend	Standard error	*df*	Lower CL	Upper CL
4.830	-0.009	0.004	247	-0.017	-0.001
5.110	-0.012	0.004	247	-0.020	-0.004
5.540	-0.016	0.005	247	-0.025	-0.007
6.940	-0.030	0.009	247	-0.048	-0.012

ESM Table 5 Simple slope analysis for the results from the stepwise regression model for hIResp with the two-way interaction between FPG and age, adjusting for BMI.

Sex	WHR	Trend	Standard error	*df*	Lower CL	Upper CL
**Male**	0.913	-0.009	0.005	248	-0.019	0.001
**Male**	0.957	-0.002	0.004	248	-0.010	0.006
**Male**	0.995	-0.004	0.005	248	-0.007	0.014
**Male**	1.180	-0.032	0.018	248	-0.004	0.068
**Female**	0.784	-0.006	0.004	248	-0.014	0.002
**Female**	0.841	-0.010	0.003	248	-0.017	-0.004
**Female**	0.885	-0.014	0.004	248	-0.021	-0.006
**Female**	1.019	-0.024	0.009	248	-0.042	-0.007

ESM Table 6 Simple slope analysis for the results from the stepwise regression model for hIResp with the three-way interaction between WHR × age × sex in female and male participants.

Sex	WHR	Trend	Standard error	*df*	Lower CL	Upper CL
**Male**	0.913	-0.008	0.005	247	-0.018	0.002
**Male**	0.957	-0.002	0.004	247	-0.010	0.006
**Male**	0.995	0.004	0.005	247	-0.006	0.014
**Male**	1.180	0.031	0.018	247	-0.005	0.067
**Female**	0.784	-0.006	0.004	247	-0.014	0.002
**Female**	0.841	-0.010	0.003	247	-0.017	-0.004
**Female**	0.885	-0.013	0.004	247	-0.021	-0.005
**Female**	1.019	-0.022	0.009	247	-0.039	-0.005

ESM Table 7 Simple slope analysis for the results from the stepwise regression model for hIResp with the three-way interaction between WHR × age × sex in female and male participants, adjusting for BMI.

	Estimate	Standard error	*t* value	*p* value
**(Intercept)**	-0.314	0.314	-1.000	0.319
**Sex**	0.571	0.532	1.074	0.285
**Age**	0.006	0.008	0.740	0.460
**VAT**	0.343	0.105	3.254	0.001
**Sex X age**	-0.015	0.012	-1.225	0.222
**Sex X VAT**	-0.314	0.142	-2.213	0.028
**Age X VAT**	-0.007	0.002	-3.096	0.002
**Sex X age X VAT**	0.007	0.003	2.522	0.013

ESM Table 8 Interaction of hIResp with age, sex and visceral adipose tissue (VAT). Coefficients of the two-sided linear regression model for hIResp and the interactions between sex, age and VAT (*n*=180, 80 missing values for VAT). Effect size adjusted $R^2 = 1.2 \cdot 10^{-1}$.

	Estimate	Standard error	*t* value	*p* value
**(Intercept)**	0.031	0.216	0.142	0.887
**Sex**	0.211	0.344	0.613	0.541
**Age**	0.000	0.005	-0.069	0.945
**IHL**	0.116	0.035	3.291	0.001
**Sex X age**	-0.005	0.008	-0.619	0.537
**Sex X IHL**	-0.112	0.050	-2.222	0.028
**Age X IHL**	-0.002	0.001	-3.524	0.001
**Sex X age X IHL**	0.003	0.001	2.606	0.010

ESM Table 9 Interaction of hIResp with age, sex and intrahepatic lipid content (IHL). Coefficients of the two-sided linear regression model for hIResp and the interactions between sex × age × IHL (*n*=177, 83 missing values for IHL). Effect size adjusted $R^2 = 1.2 \cdot 10^{-1}$.

	Estimate	Standard error	*t* value	*p* value
**(Intercept)**	-0.403	0.360	-1.118	0.265
**Sex**	0.558	0.533	1.047	0.297
**Age**	0.005	0.008	0.605	0.546
**VAT**	0.306	0.129	2.378	0.018
**BMI**	0.005	0.011	0.506	0.614
**Sex X age**	-0.015	0.012	-1.191	0.235
**Sex X VAT**	-0.294	0.147	-1.996	0.048
**Age X VAT**	-0.006	0.002	-2.619	0.010
**Sex X age X VAT**	0.007	0.003	2.372	0.019

ESM Table 10 Coefficients of the two-sided linear regression model for hIResp and the interactions between sex × age × VAT, adjusting for BMI (*n*=180).

	Estimate	Standard error	*t* value	*p* value
**(Intercept)**	-0.229	0.291	-0.788	0.432
**Sex**	0.196	0.343	0.571	0.568
**Age**	-0.002	0.005	-0.383	0.702
**IHL**	0.101	0.037	2.720	0.007
**BMI**	0.011	0.008	1.335	0.184
**Sex X age**	-0.004	0.008	-0.514	0.608
**Sex X IHL**	-0.103	0.051	-2.038	0.043
**Age X IHL**	-0.002	0.001	-3.044	0.003
**Sex X age X IHL**	0.002	0.001	2.418	0.017

ESM Table 11 Coefficients of the two-sided linear regression model for hIResp and the interactions between sex × age × IHL, adjusting for BMI (*n*=177).

Sex	VAT	Trend	Standard error	*df*	Lower CL	Upper CL
**Male**	3.460	-0.008	0.005	172	-0.018	0.003
**Male**	5.620	-0.007	0.005	172	-0.017	0.003
**Male**	7.540	-0.006	0.007	172	-0.020	0.008
**Male**	14.820	-0.003	0.019	172	-0.040	0.034
**Female**	2.240	-0.009	0.004	172	-0.018	-0.001
**Female**	3.400	-0.017	0.004	172	-0.024	-0.009
**Female**	4.590	-0.025	0.005	172	-0.034	-0.015
**Female**	8.400	-0.050	0.012	172	-0.073	-0.026

ESM Table 12 Simple slope analysis for the results from the stepwise regression model for hIResp with the three-way interaction between VAT × age × sex in female and male participants.

Sex	IHL	Trend	Standard error	*df*	Lower CL	Upper CL
**Male**	2.300	-0.005	0.005	169	-0.014	0.005
**Male**	5.420	-0.004	0.004	169	-0.012	0.004
**Male**	11.130	-0.003	0.006	169	-0.014	0.008
**Male**	29.620	-0.0001	0.017	169	-0.034	0.034
**Female**	1.890	-0.005	0.004	169	-0.013	0.003
**Female**	3.570	-0.009	0.004	169	-0.016	-0.002
**Female**	7.450	-0.018	0.004	169	-0.026	-0.011
**Female**	22.400	-0.054	0.012	169	-0.079	-0.030

ESM Table 13 Simple slope analysis for the effects of the stepwise regression model for hIResp with the three-way interaction between IHL × age × sex in female and male participants.

**Sex**	**VAT**	**Trend**	**Standard error**	***df***	**Lower CL**	**Upper CL**
**Male**	3.460	-0.008	0.005	171	-0.018	0.003
**Male**	5.620	-0.006	0.005	171	-0.017	0.004
**Male**	7.540	-0.005	0.007	171	-0.020	0.009
**Male**	14.820	-0.001	0.019	171	-0.039	0.038
**Female**	2.240	-0.009	0.004	171	-0.017	-0.001
**Female**	3.400	-0.016	0.004	171	-0.024	-0.008
**Female**	4.590	-0.023	0.006	171	-0.034	-0.012
**Female**	8.400	-0.046	0.013	171	-0.073	-0.020

ESM Table 14 Simple slope analysis for the results from the stepwise regression model for hIResp with the three-way interaction between VAT × age × sex in female and male participants, adjusting for BMI.

**Sex**	**IHL**	**Trend**	**Standard error**	***df***	**Lower CL**	**Upper CL**
**Male**	2.300	-0.005	0.005	168	-0.015	0.004
**Male**	5.420	-0.004	0.004	168	-0.013	0.004
**Male**	11.130	-0.003	0.006	168	-0.014	0.008
**Male**	29.620	0.002	0.017	168	-0.033	0.036
**Female**	1.890	-0.006	0.004	168	-0.014	0.002
**Female**	3.570	-0.010	0.004	168	-0.017	-0.003
**Female**	7.450	-0.018	0.004	168	-0.025	-0.010
**Female**	22.400	-0.050	0.013	168	-0.075	-0.025

ESM Table 15 Simple slope analysis for the results from the stepwise regression model for hIResp with the three-way interaction between IHL × age × sex in female and male participants, adjusting for BMI.

	**Estimate**	**Standard error**	***t* value**	***p* value**
**(Intercept)**	-0.062	0.494	-0.126	0.900
**Sex**	-0.200	0.764	-0.262	0.793
**Age**	-0.007	0.011	-0.688	0.493
**SAT**	0.033	0.027	1.235	0.218
**Sex X age**	0.011	0.017	0.655	0.514
**Sex X SAT**	0.005	0.052	0.099	0.921
**Age X SAT**	0.000	0.001	-0.438	0.662
**Sex X age X SAT**	0.000	0.001	-0.195	0.846

ESM Table 16 Coefficients of the two-sided linear regression model for hIResp and the interactions between sex × age × SAT (*n*=180, 80 missing values for SAT).

	**Estimate**	**Standard error**	***t* value**	***p* value**
**(Intercept)**	-0.088	0.525	-0.169	0.866
**Sex**	-0.216	0.773	-0.279	0.780
**Age**	-0.008	0.011	-0.698	0.486
**SAT**	0.031	0.030	1.065	0.288
**BMI**	0.002	0.014	0.151	0.880
**Sex X age**	0.011	0.017	0.665	0.507
**Sex X SAT**	0.006	0.052	0.111	0.912
**Age X SAT**	0.000	0.001	-0.434	0.664
**Sex X age X SAT**	0.000	0.001	-0.210	0.834

ESM Table 17 Coefficients of the two-sided linear regression model for hIResp and the interactions between sex × age × SAT, adjusting for BMI (*n*=180).

	Trend	Standard error	*df*	Lower CL	Upper CL
**WHR**					
**premenopausal**	0.794	0.775	159	-0.737	2.33
**postmenopausal**	-3.539	1.192	159	-5.893	-1.18
**VAT**					
**premenopausal**	0.113	0.048	104	0.018	0.201
**postmenopausal**	-0.074	0.048	104	-0.168	0.020
**IHL**					
**premenopausal**	0.050	0.017	101	0.017	0.083
**postmenopausal**	-0.030	0.012	101	-0.054	-0.006

ESM Table 18 Slopes from the linear regression model for hIResp and waist-to-hip ratio (WHR) or VAT or IHL according to menopausal status (*n*=163 (WHR), *n*=108 (VAT), *n*=105 (IHL)).

	Trend	Standard error	*df*	Lower CL	Upper CL
**WHR**					
**premenopausal**	0.219	0.845	158	-1.450	1.890
**postmenopausal**	-3.795	1.195	158	-6.160	-1.430
**VAT**					
**premenopausal**	0.059	0.070	103	-0.080	0.198
**postmenopausal**	-0.095	0.051	103	-0.197	0.007
**IHL**					
**premenopausal**	0.043	0.018	100	0.008	0.078
**postmenopausal**	-0.032	0.012	100	-0.056	-0.008

ESM Table 19 Slopes from the linear regression model for hIResp and waist-to-hip ratio (WHR) or VAT or IHL according to menopausal status, adjusting for BMI (*n*=163 (WHR), *n*=108 (VAT), *n*=105 (IHL)).

**Sex**	**Cluster (high-risk, low-risk)**	**Trend**	**Standard error**	***df***	**Lower CL**	**Upper CL**
**Female**	**Low-risk**	-0.004	0.005	198	-0.014	0.007
**Male**	**Low-risk**	-0.007	0.005	198	-0.017	0.003
**Female**	**High-risk**	-0.018	0.004	198	-0.026	-0.009
**Male**	**High-risk**	-0.004	0.007	198	-0.017	0.010

ESM Table 20 Slopes from the two-sided linear regression model for hIResp and the interactions between sex × age × Tübingen Prediabetes Cluster category (high-risk or low-risk; n=206).

**Predictors**	**Estimate**	**CI**	***p* value**
**(Intercept)**	-0.040	-1.190 – 1.120	0.950
**Sex**	0.690	-0.780 – 2.170	0.355
**Age**	0.000	-0.020 – 0.030	0.886
**Cluster 2**	0.140	-1.290 – 1.580	0.843
**Cluster 3**	1.460	-1.530 – 4.450	0.338
**Cluster 4**	0.540	-0.820 – 1.900	0.438
**Cluster 5**	0.920	-0.590 – 2.430	0.231
**Cluster 6**	0.690	-0.580 – 1.970	0.285
**Sex X age**	-0.020	-0.060 – 0.020	0.297
**Sex X cluster 2**	-0.760	-2.580 – 1.050	0.407
**Sex X cluster 3**	-1.920	-5.320 – 1.470	0.265
**Sex X cluster 4**	-1.020	-2.950 – 0.910	0.297
**Sex X cluster 5**	-0.640	-2.810 – 1.520	0.558
**Sex X cluster 6**	-2.020	-4.120 – 0.090	0.060
**Age X cluster 2**	-0.000	-0.030 – 0.030	0.894
**Age X cluster 3**	-0.030	-0.090 – 0.020	0.234
**Age X cluster 4**	-0.010	-0.040 – 0.020	0.519
**Age X cluster 5**	-0.020	-0.050 – 0.010	0.197
**Age X cluster 6**	-0.020	-0.050 – 0.010	0.245
**Sex X age X cluster 2**	0.010	-0.030 – 0.060	0.506
**Sex X age X cluster 3**	0.050	-0.020 – 0.110	0.152
**Sex X age X cluster 4**	0.030	-0.020 – 0.070	0.248
**Sex X age X cluster 5**	0.020	-0.020 – 0.070	0.348
**Sex X age X cluster 6**	0.050	0.000 – 0.100	0.035

ESM Table 21 Coefficients of the linear regression model for hIResp and the interactions between sex × age × Tübingen Prediabetes Cluster (*n*=206).

	**Estimate**	**Standard error**	***t* value**	***p* value**
**(Intercept)**	89.240	6.352	14.049	0.000
**Sex**	10.184	11.187	0.910	0.364
**Age**	0.144	0.138	1.043	0.298
**Sex X age**	-0.484	0.235	-2.059	0.041

ESM Table S22 Coefficients of the two-sided linear regression model for food craving (trait) and the interactions between sex and age (*n*=227).

**Sex**	**Trend**	**Standard error**	***df***	**Lower CL**	**Upper CL**
**female**	0.144	0.138	223	-0.128	0.417
**male**	-0.339	0.190	223	-0.714	0.035

ESM Table 23 Slopes from the two-sided linear regression model for food craving (trait) according to sex (*n*=227).

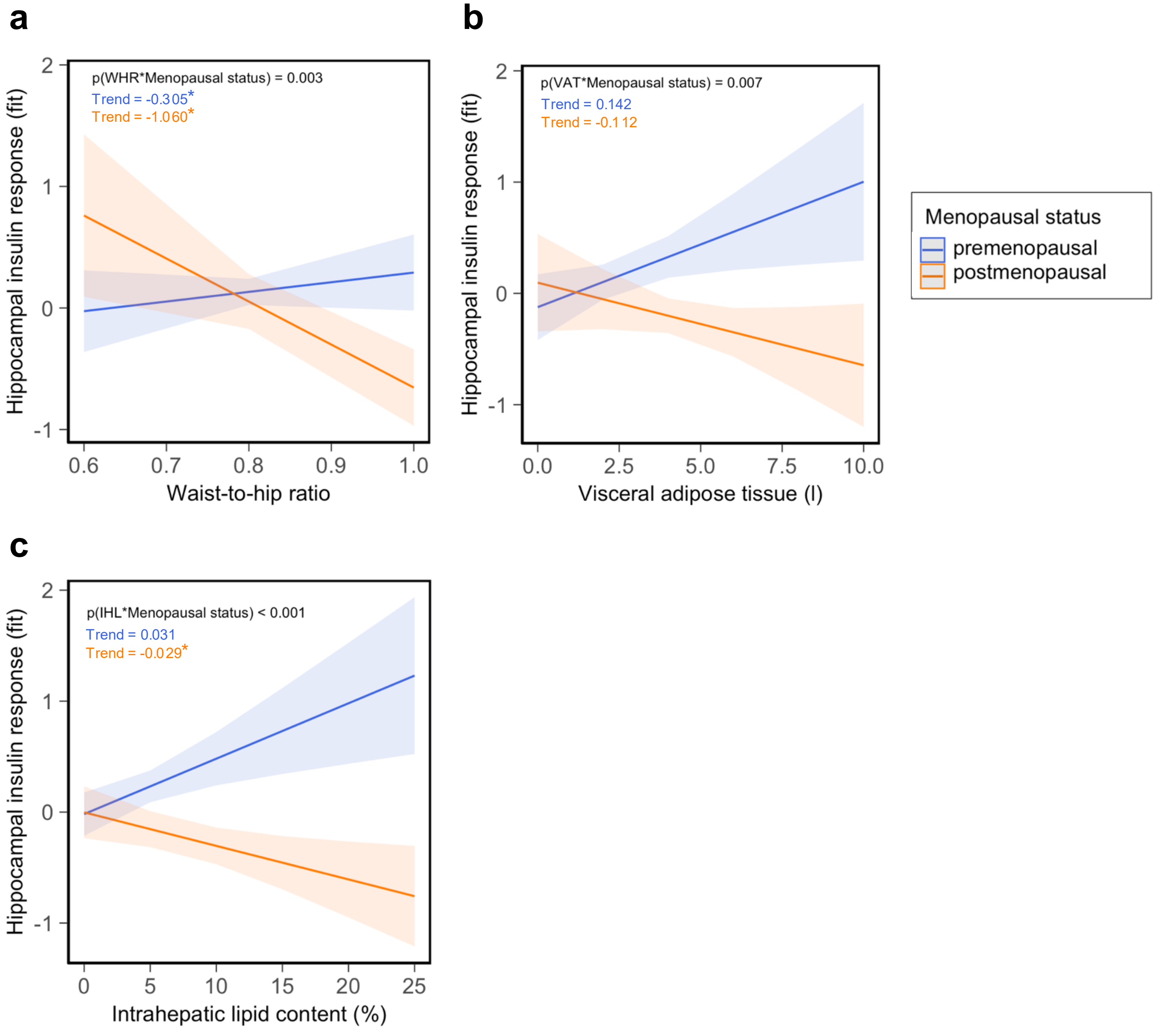

ESM Fig. 1 Association between hIResp and body fat distribution according to menopausal status. (**a**) Waist-to-hip ratio (*n*=163, 1 female with hormonal replacement therapy (HRT) was excluded, 1 missing value for WHR), (**b**) visceral adipose tissue (*n*=108, 1 female with HRT, 56 missing values for VAT) and (**c**) intrahepatic lipid content (*n*=105, 1 female with HRT, 59 missing values for IHL) show significant interactions with menopausal status on hIResp. Shaded areas around the line depict the 95% confidence interval. An asterisk (*) denotes a significant simple slope, defined as a 95% CI that did not include 0.

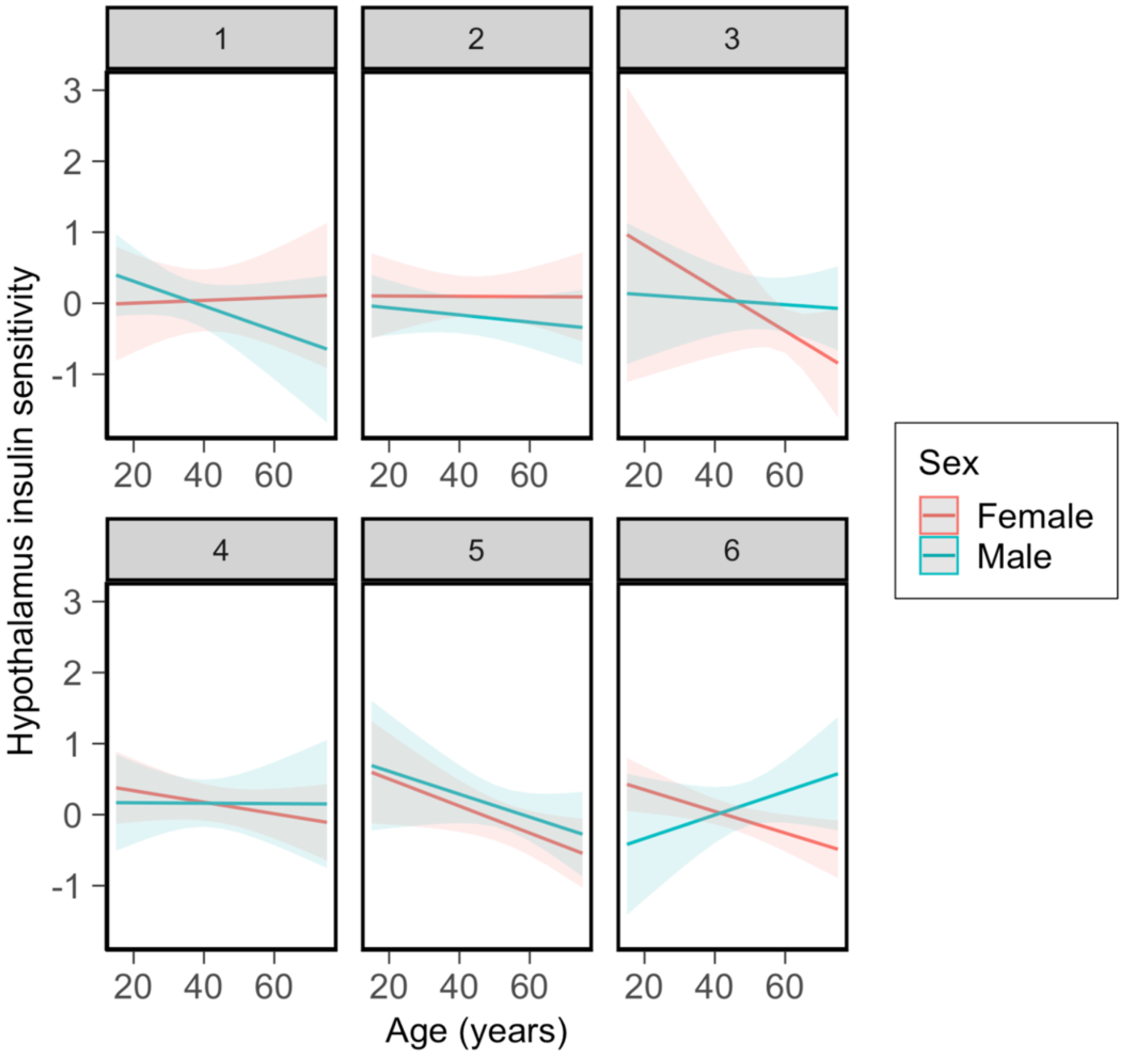

ESM Fig. 2 Age-dependent hIResp in Tübingen Prediabetes Clusters in women and men. Clusters are divided into low-risk (cluster 1, 2 and 4) and high-risk (clusters 3, 5 and 6) separately. Plots show the effects of a two-way interaction between cluster × sex on hIResp using a two-sided linear regression model (n=206). Slopes can be found in Table S21. Shaded areas around the line depict the 95% confidence interval

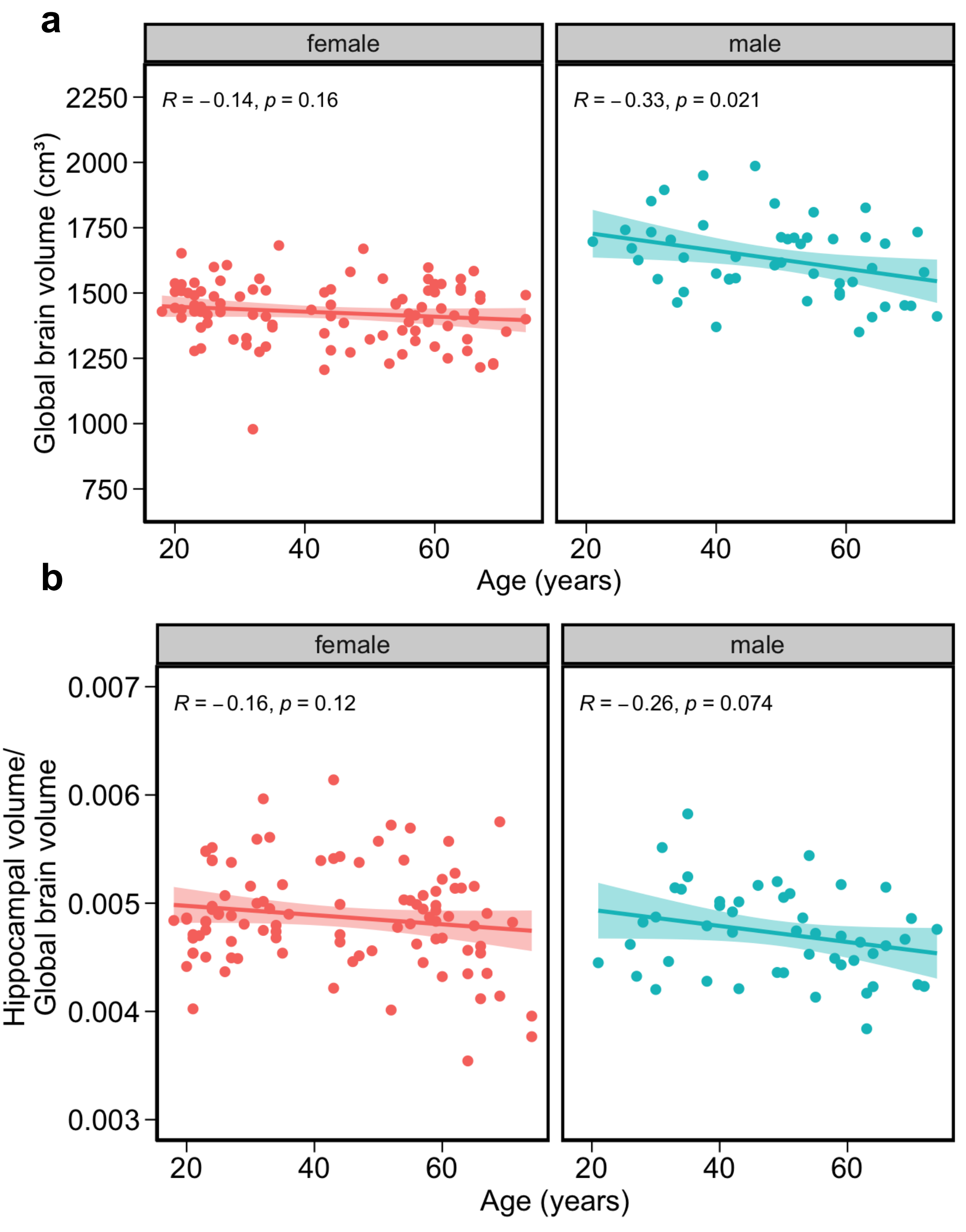

ESM Fig. 3 Global brain and hippocampal volume in women and men. (**a**) Global brain volume and (**b**) normalized hippocampal volume significantly correlates with age in men but not women (n=150, 110 missing values for brain volume measurements).

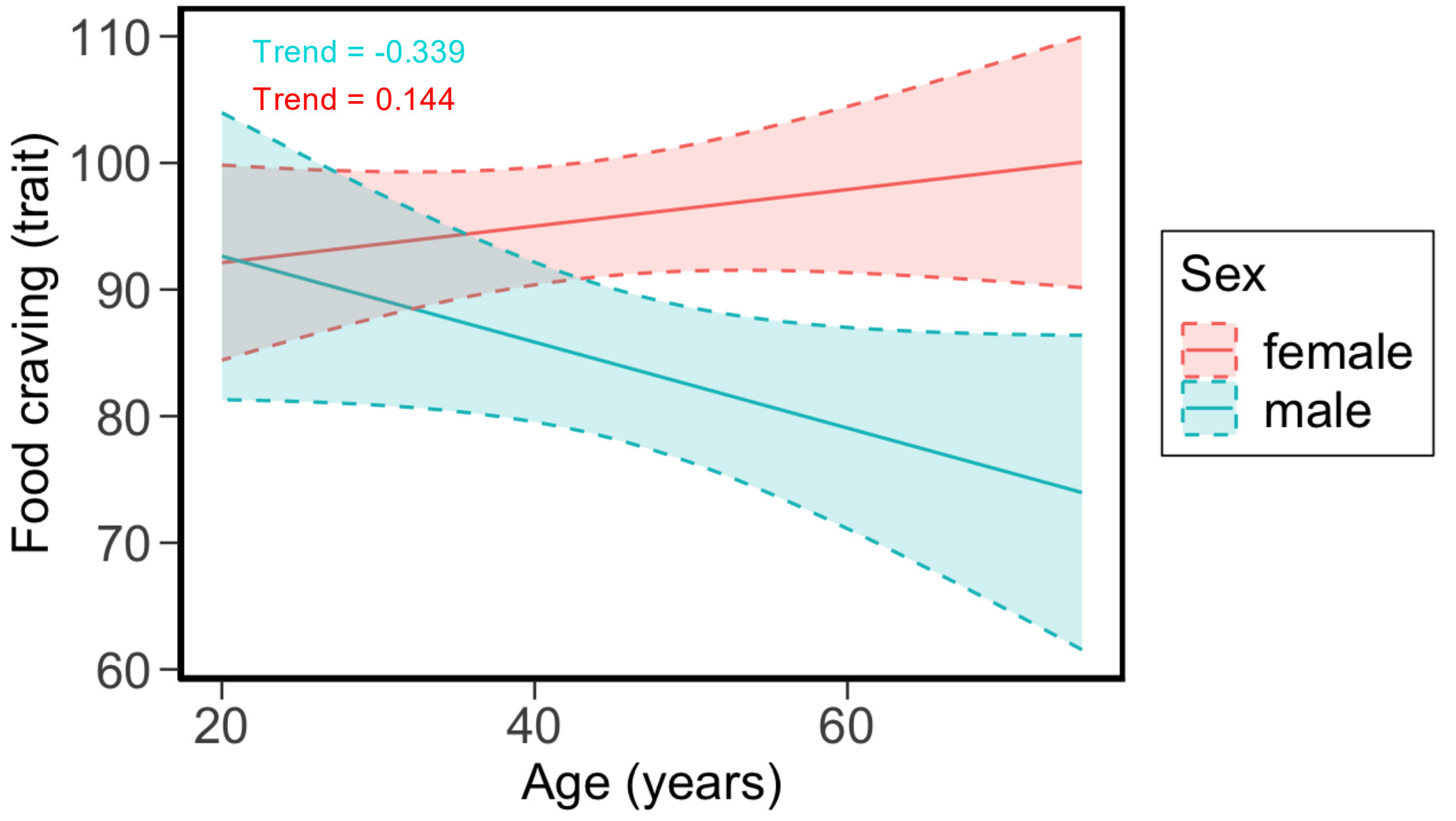

ESM Fig. 4 Food craving score in female and male participants. Plots show the effects of a two-way interaction between age × sex on the food-craving sum score using a two-sided linear regression model (*n*=227, 33 missing values for FCQ-T). Older women show higher food craving with increasing age than men. Shaded areas around the line depict the 95% confidence interval.

Abbreviations: BMI, body mass index; CI, confidence interval; CL, confidence limit; FCQ-T, Food Cravings Questionnaire–Trait; FPG, fasting plasma glucose; GVIF, generalised variance inflation factor; hIResp, hippocampal insulin response HRT, hormonal replacement therapy; IHL, intrahepatic lipid content; R^2, coefficient of determination; SAT, subcutaneous adipose tissue; VAT, visceral adipose tissue; WHR, waist-to-hip ratio.
